# Evidence of interparticle chylomicron “bridging” in mouse mesenteric lymph after a lipid bolus

**DOI:** 10.1016/j.jlr.2025.100946

**Published:** 2025-11-15

**Authors:** Khaga Raj Neupane, Alexander Karakashian, Min Liu, Scott M. Gordon

**Affiliations:** 1 Saha Cardiovascular Research Center, University of Kentucky, Lexington, KY, USA; 2 Department of Pathology and Laboratory Medicine, University of Cincinnati, Cincinnati, OH, USA; 3 Department of Physiology, University of Kentucky, Lexington, KY, USA

**Keywords:** apolipoprotein B, chylomicron, dietary lipid, intestine, lipoprotein, mesenteric lymph

Detailed examination of chylomicrons in plasma is challenging because these particles are rapidly hydrolyzed. In this study, we examined the morphology of newly secreted chylomicrons prior to entry into the circulation by collecting mesenteric lymph from wild-type mice while fasting and after a duodenal lipid bolus. Lymph was examined by negative-stain transmission electron microscopy (Figure 1). Fasting lymph contained predominantly small lipoproteins. Following duodenal Intralipid™ infusion, we observed large chylomicrons. Notably, many of these particles appeared connected by bridge-like structures (yellow arrows) suggestive of lipoprotein remodeling occurring in the intestinal lymph prior to their entry in the circulation.

**Fig. 1:**
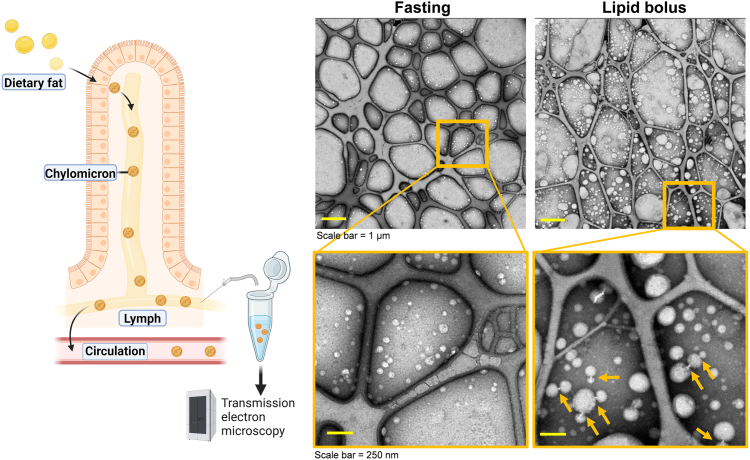
**Bridge-like structures observed linking chylomicrons in postprandial intestinal lymph.** TEM analysis of fresh, never-frozen mesenteric lymph collected via conscious lymph fistula before and 3 hours after administration of a duodenal lipid bolus. Images reveal the presence of distinct docking sites linking chylomicron particles (yellow arrows) in post-lipid bolus samples, sometimes forming multi-particle complexes. We speculate that these are protein bridges that facilitate the exchange of lipids between particles.

Methods: Fresh, never-frozen, mesenteric lymph collected by conscious lymph fistula (1) was applied to copper EM grids (400-mesh; Ted Pella) and stained with 2% uranyl acetate. Electron micrographs were acquired using a Talos F200X transmission electron microscope (Thermo Fisher Scientific).

## Conflict of interest

The authors declare that they have no conflicts of interest related to the contents of this article.
